# Data on fluoride concentration levels in cold and warm season in rural area of Shout (West Azerbaijan, Iran)

**DOI:** 10.1016/j.dib.2017.10.012

**Published:** 2017-10-10

**Authors:** Farzaneh Baghal Asghari, Ali Akbar Mohammadi, Zahra Aboosaedi, Mehdi Yaseri, Mahmood Yousefi

**Affiliations:** a Department of Environmental Health Engineering, Tehran University of Medical Sciences, Tehran, Iran; b Department of Environmental Health Engineering, Neyshabur University of Medical Sciences, Neyshabur, Iran; c Department of Epidemiology and Biostatistics, School of Public Health, Tehran University of Medical Sciences, Tehran, Iran; d Students Research Committee, Neyshabur University of Medical Sciences, Neyshabur, Iran

**Keywords:** Shout, Fluoride, Drinking water, Season

## Abstract

The aim of this study was to determine the concentration of fluoride in drinking water, the distribution system, in 22 villages in Shout (A city in West Azerbaijan province). Sampling of springs and underground water was carried out in two warm and cold seasons. Fluoride concentration were determined through spectrophotometer with a model, DR/5000.The fluoride concentration were compared with Iranian standards and WHO guidelines for drinking water.

**Specifications Table**

**Table:** 

Subject area	Water chemistry
More specific subject area	Water fluoride
Type of data	Table, figure
How data was acquired	Spectrophotometer (DR/5000, Hach)
Data format	Raw, analyzed
Experimental factors	Water samples were stored in a dark place at room temperature in their original sealed plastic containers (200 mL) until the fluoride analysis was made
Experimental features	Determine the concentration levels of fluoride
Data source location	Shout area, West Azerbaijan province, Iran
Data accessibility	Data are included in this article

**Value of the data**

• The main health outcomes of fluoride, including dental and skeletal fluorosis.
• The Iranian standard of fluoride in drinking water is based on the maximum annual temperature of the area because of temperature impact on water consumption.
• In cooler areas, such as West in Northern Iran, the consumption of drinking water is lower, thus higher fluoride concentrations in drinking water are required.
• Fluoridation of drinking water in rural areas with less than the WHO optimum value is recommended.
• Based on the data, defluoridation of drinking water could be recommended in fluorotic rural areas.

## 1 Data

Based on Table 1, the average concentration of fluoride in warm and cold seasons (in spring and groundwater) were 0.01–3 and 0.01–4 mg/l, respectively. According Iranian standard (1053IR) World Health Organization (WHO), in 57.9% and 18.2%of samples fluoride concentration in warm and cold season was less than the permissible limit respectively.

**Table 1:** Mean fluoride concentrations (mg/L) in drinking water of rural areas of Shout region according to places and seasons.

**Village**	**Source**	**Warm season**		**Cold season**		**Warm season**				**Cold season**			
		**Average**		**Average**		**Fluoride concentration**				**Fluoride concentration**			
		**T(°C)**	**pH**	**T(°C)**	**pH**	**Mean**	**Min**	**Max**	**Sd. ev**	**Mean**	**Min**	**Max**	**Sd. ev**
Azimkandi	Spring	23	7.8	19	7.6	1.39	1.36	1.42	0.03	2	1.8	2.4	0.27
Maranglou	Spring	23	8.9	19	8.9	0.44	0.42	0.46	0.02	1.97	1.94	2	0.03
Injaghadim	Spring	23	8.8	17	8.2	0.01	0	0.03	0.02	1.18	1.15	1.24	0.04
Kolos	Well	24	7.9	19	7.9	1.43	1.36	1.52	0.07	3.1	2.75	3.4	0.30
Shorboulagh	Well	23	7.9	18	7.9	1.52	1.39	1.62	0.10	4	3.9	4.1	0.08
Karimkandi	Well	23	7.8	19	7.8	0.38	0.32	0.45	0.05	0.01	0	0.04	0.02
Fatah	Well	23	8	19	7.8	1.01	0.68	1.41	0.35	1	0.8	1.3	0.21
Molaahmad	Well	23	7.6	19	7.6	3	2.7	3.2	0.24	0.09	0.06	0.11	0.02
Pivasha	Well	23	7.8	19	7.8	0.37	0.3	0.44	0.07	1.25	1.22	1.31	0.04
Gara eyagh	Well	23	7.9	19	7.9	0.45	0.34	0.54	0.09	1.86	1.83	1.91	0.04
Khook	Well	22	7.8	19	7.5	1.38	1.37	1.4	0.01	0.79	0.68	0.89	0.10
Kesharkhi	Well	23	7.8	19	7.8	1.50	1.48	1.52	0.02	1.8	1.78	1.81	0.01
Garazamin	Well	23	7.9	17	7.8	0.20	0.1	0.28	0.08	1.8	1.42	2.3	0.37
Moukhor	Well	23	7.8	19	7.8	0.1	0.06	0.14	0.04	1	0.75	1.25	0.26
Yolagaldi	Well	23	7.8	18	7.8	1.2	1	1.4	0.18	3.5	3.3	3.7	0.18
Margan	Well	23	8.2	19	8.2	1.3	1.08	1.52	0.24	3	2.7	3.2	0.24
Tazakand	Well	23	7.8	19	7.5	0.2	0.12	0.26	0.06	0.2	0.14	0.25	0.05
Geday	Well	23	7.8	19	7.8	1	0.7	1.4	0.32	1.11	1.04	1.18	0.06
Khilajajam	Well	22	7.9	18	7.4	2.5	1.6	3.1	0.73	1	0.79	1.15	0.15
Khezrlou	Well	23	7.9	19	7.8	0.2	0.1	0.26	0.07	1.2	1.06	1.45	0.18
Toura	Well	23	7.8	19	7.9	0.3	0.16	0.36	0.09	1.8	1.62	2.1	0.21
Gabanbasan	Well	23	7.8	19	7.8	1	0.6	1.3	0.32	0.9	0.84	0.98	0.07
1053IR Standard						0.7				1.2			
WHO Standard						0.8				1.2			

## 2 Experimental design, materials and methods

### 2.1 Study area description

**Fig. 1:**
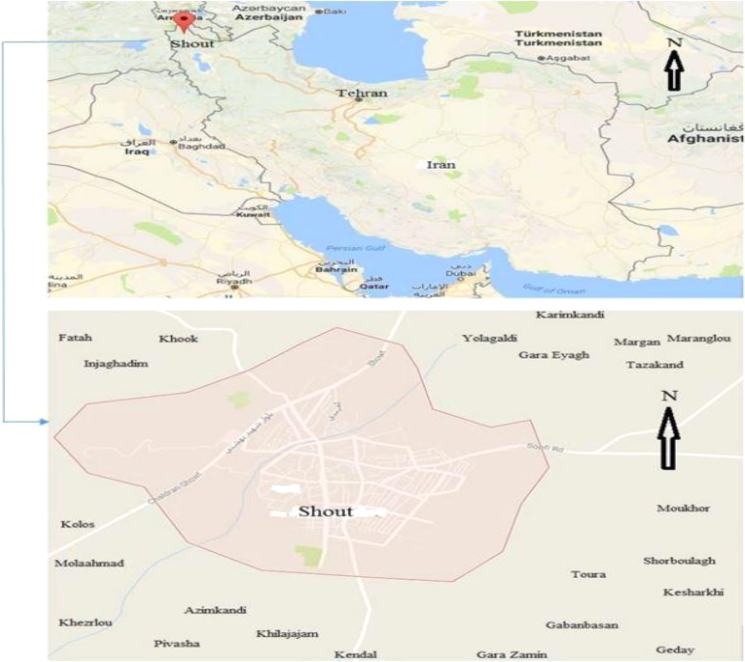
The map and location of sampling villages in Shout city, West Azerbaijan, Iran.

West Azerbaijan province is one of the 31 provinces of Iran. Shout is a city in West Azerbaijan province, Iran that coordinates is: 39°13′09″N 44°46′12″E﻿/39.21917 °N 44.77000°E. 22 villages of Shout were selected as sampling points (Fig. 1).

### 2.2 Sample collection and analytical procedures

The samples were collected from 22 points in the rural areas of Shout in the warm and cold seasons through using census method (6 samples from each village, 3 samples in the warm season and 3 samples in the cold season). Transportation (PE containers, 200 ml) and storage of samples were carried out in accordance with standard methods of water and wastewater treatment [1], [2], [3], [4], [5], [6], [7], [8]. To determine the residual fluoride concentration of spectrophotometer UV–vis DR-500 (SPAND method was used. Finally, the concentration of fluoride was compared with Iranian and international (WHO guideline)) standards [9], [10].
