# Supplementary material for: Data on fluoride concentration levels in cold and warm season in rural area of Shout (West Azerbaijan, Iran)
Source: Data Brief. 2017 Oct 10;15:528–31. doi: 10.1016/j.dib.2017.10.012 (PMC5651490; doi:10.1016/j.dib.2017.10.012)
Supplement: Supplementary file 1 — Supplementary material [file mmc1.docx]

**Competing interests**

**The authors declare no Conflict of Interest.**
